# Future of Endoscopy in Inflammatory Bowel Diseases (IBDs)

**DOI:** 10.7759/cureus.29567

**Published:** 2022-09-25

**Authors:** Laksh S Agrawal, Sourya Acharya, Samarth Shukla, Yash C Parekh

**Affiliations:** 1 Department of Medicine, Jawaharlal Nehru Medical College, Datta Meghe Institute of Medical Sciences (Deemed to be University), Wardha, IND; 2 Department of Pathology, Jawaharlal Nehru Medical College, Datta Meghe Institute of Medical Sciences (Deemed to be University), Wardha, IND; 3 Medicine and Surgery, Jawaharlal Nehru Medical College, Datta Meghe Institute of Medical Sciences (Deemed to be University), Wardha, IND

**Keywords:** endopathology, imaging, gastrointestinal lesions, inflammatory bowel disease, endoscopy

## Abstract

Gastrointestinal (GI) endoscopy has transformed over the years in scope, safety, accuracy, acceptability, and cost effectiveness of the clinical practice. There has been a reduction in the superiority of the endoscopic devices as innovations have taken place and increased the diagnostic values with certain limitations. There are particular difficulties in striking a balance between the development of new technology and the device's acceptance. The wide use of endoscopy for investigating GI lesions and diagnosis has led to an increase in more advanced methods and their broad application. It can simultaneously diagnose pre-malignant and malignant lesions, and newer interventions have made the biopsy specimen uptake possible. In this review article, we focus on the more recent roles, indications, applications, and usage of the innovative methods of endoscopy.

## Introduction and background

Endoscopy

Endoscopy is used for the diagnosis and surveillance of pre-malignant gastrointestinal (GI) lesions [1]. It provides an accurate and easy method for the diagnosis of GI lesions. It has facilitated the diagnosis of lesions by developing high resolution, which can differentiate between the inflamed and neoplastic tissues on visual inspection. There are different types of endoscopes used for the diagnostic purpose of GI tract (GIT) lesions. It is used for the visualization of the internal organs and guidance of surgical procedures. The endoscopes used include rigid endoscopes, flexible endoscopes, and capsule endoscopes, among others. Each endoscope is associated with different use and application: rigid endoscopes are used in surgical procedures; flexible endoscopes are used to inspect the lumen of the small intestine; and capsule endoscopes are used for inspection of the GIT. The process of chromoendoscopy requires the installation of dye inside the GIT for visualization with a fiber-optic endoscope. The purpose of this procedure is to enhance the characterization of tissues, as dyes can be used for different purposes. These endoscopes are traditionally used in the present, and newer practices have evolved in the usage them following more recent developments in the procedures to make them more concise and minimalistic.

GI lesion with associated anatomy

GI lesions present with diseases defined as irritation, break or an inflammation in the GI mucosa. It can develop in different areas of the GI system. The upper GIT and lower GIT portions of the system are separated. The mouth, esophagus, stomach, and duodenum constitute the upper GIT. The small and large intestines and the anus constitute the lower GIT. There are numerous illnesses that incorporate the entire system. The ignorance of symptoms can lead to lesions varying in severity from benign to malignant. Inflammatory bowel disease (IBD) entails two diseases: ulcerative colitis (UC) and Crohn's disease (CD). These diseases sometimes result in extensive damage to the mucosa of the GIT. Clinical and laboratory data, radiographic anomalies, histology, and endoscopy are all included in a multifactorial approach for the differential diagnosis of both illnesses [2]. Esophagogastroduodenoscopy, flexible sigmoidoscopy, and colonoscopy have all been used to make the diagnosis in these patients [3]. IBD is a risk factor for developing colorectal cancer. A cumulative incidence for colorectal cancer has been conducted in the form of a meta-analysis which states 2% at 10 years, 8% at 20 years and 18% after 30 years of the disease [4]. The endoscopic technologies have advanced to endoscopic ultrasound, capsule endoscopy, balloon-assisted enteroscopy, and other new interventions in the field of IBD.

CD and UC are persistent inflammatory gut infections that are becoming more common all over the world. They have a remarkable impact on overall prosperity, social functioning, and the utilization of medical resources [5,6]. The analysis of IBD is difficult for doctors, being founded on various components like clinical information, biochemical qualities, radiology, endoscopy, and pathology [7]. Amid them, endoscopy addresses a foundation in the finding and looks into CD and UC [8,9].

Over the last few years, the idea of endoscopy has developed from a customary one to a groundbreaking thought in light of computerized reasoning (simulated intelligence). Artificial intelligence (AI) is characterized as any machine that has mental capabilities mirroring people for critical thinking or learning [10]. AI has as of now been tried in a few fields of endoscopy, for example, in the discovery of Barrett's esophagus or the assessment of carcinoma location rate in the course of colonoscopy [11-13].

Etiology of IBD

Genetic Variation

The disease predispostion is seen on chromosomes 16q,12p,6p,14q and 5q. Caspase-associated recruitment domain containing protein 15 (CARD15) on chromosome 16q is expressed by cells in the intestinal mucosa, resulting in loss of function and a 50 times risk of developing CD. In a recent study, the number of IBD-associated gene loci has increased to 163 as a result of recent investigations, of which 110 are linked to both diseases, 30 CD and 23 UC [14].

Alcohol and Smoking

The role of smoking and alcohol has led to an increase in the development of IBDs. In cases of UC, smoking decreases the risk, whereas in CD, smoking increases the risk and is associated with an increased rate of post-operative disease [15].

Malabsorption and Intestinal Flora Impairment

It is suspected that various microorganisms such as mycobacterium paratuberculosis, salmonella, shigella, helicobacter, clostridia, bactericides, escherichia, and measles virus have led to the initiation of inflammatory mediators. The impaired epithelial transport and integrity are related to malabsorption, which reduces the absorption of nutrients [16]. In addition, the alterations in the mucosal epithelium can cause loss of blood, proteins and electrolytes with fluid. 

Psychological Factors

Depression and anxiety are two mood elements of stress that may be very important in modulating the progression of IBD [17].

Decreased Effect of the Immune System

The activation of CD4+ T cells secreting cytokines, which suppresses the inflammation in the gut wall, is impaired. In IBD, the immune mechanism is defective and results in uncontrolled inflammation.

Drugs

Aspirin and non-steroidal anti-inflammatory drugs (NSAIDs) have an effect that increases the risk of IBD. However, a recent study found no association between aspirin dose, duration, and frequency, but prolonged use with a high dosage has been a risk factor for IBD [18]. 

Assessment of GIT Lesions

Endoscopy in the Diagnosis of the Disease

IBD patients require an initial colonoscopy with intubation, while examining the terminal ileum [19]. In UC, the diagnostic modality remains the same, whereas, for CD, the diagnostic modality changes according to the GIT region involved. The colon and terminal ileum can be visualized directly. If the patient has IBD, two biopsies were collected from five sites in the ileum and the rectum [20]. However, multiple biopsies can be collected from the ileum and the rectum. The standard endoscopy can be performed on a sedated patient. It can be diagnosed in the upper or the lower GIT from a white light endoscope that allows imaging, biopsies, and treatment in a particular session [21]. A few studies have reported frequent missing of the GIT lesion consisting IBD while using the standard endoscopy method in the early onset of the disease [22-23]. The more recent techniques, such novel endoscopy needed for better technologies, present with greater accuracy, enhanced sampling, and less invasive treatments, which can result in early detection and treatment of the lesions.

## Review

Older interventions

White-Light Endoscopy

The white light illumination with a conventional endoscope is a gold standard for assessing the GIT lesions. The advancements in image sensors and optics, which have higher pixel densities and magnification, have benefited this endoscope. Advanced imaging comprises a 150-fold optical magnification expansion as well as several methods to enhance the endoscope's mucosal features. If improvements are made in the optical field of view, resolution, and three-dimensional (3D) imaging capability for white-light endoscopes (WLEs), they can be used for better diagnostic purposes. Currently, they can support 2K for video acquisition with a field of view of 140-170 degrees. If improvements are made, then visualization of lesions can be detected and assessed in an improved manner, and the field of view can be increased to 245-330 degrees [24-26]. The laparoscopic applications and animal models have a resolution of 4K or 8k, and 3D imaging capabilities [27-29] can demonstrate the detailed mucosal and increase the surgical maneuver of the GIT.

Dye-Based Chromoendoscopy

This method has been used for enhancing the mucosal surface pattern morphologies and represents one of the oldest techniques in endoscopy [30]. The two types of dyes used clinically are methylene blue and Lugol's iodine. Indigo carmine acts as a contrasting dye. Methylene blue is absorbed in the epithelial cells of the small intestine and provides contrast enhancement, but concerns are increased for DNA damage [31]. The normally stored Lugol's iodine primarily binds to glycogen. Indigo carmine enhances the topology of mucosal by filling in the cervices, pits and ridges. The imaging source depends on the uptake mechanisms of the tissue dye. This method lacks newer improvisation, which is seen in virtual chromoendoscopy. In chromoendoscopy, it is safe and relatively inexpensive, which can enhance mucosal contrast. Still, it requires the quality and uniformity of spraying of the dye and expertise in the field.

Ultra-Thin Endoscopy

When compared to traditional endoscopes, ultra-thin endoscopes have many advantages like being safe, affordable, and simple to use [32]. They come in disposable and transportable forms, changing the way practical work is done. They are similar to normal endoscopes but with shaft sizes that range from 6 mm to less. There are no drawbacks or limitations of this device. The biopsy conducted by this endoscope is compared to the standard endoscope for the diagnostic yield for dysplasia [33].

*Capsule Endoscopy*

The US Food and Drug Administration (FDA) approved capsule endoscopes in 2001 to provide safe and easy access to the GIT. It has evolved over two decades for the management of bowel disease [34]. Their diagnostic value is 71%, which is reliable for CD, and provide lowered imaging quality and patency [35,36]. The wireless data transmission is via wireless radio telemetry or electric field propagation [37]. The upper part of GIT is difficult to capture due to its anatomy but they can capture 35 frames/second using the pill cam capsule. The lower part of GIT can detect polyps with incomplete colonoscopy [38]. The disadvantage lies in the lack of locomotion which can limit the usage of the imaging quality in organs.

A comparison is provided between WLE, ultra-thin endoscope, and capsule endoscope in Table 1.

**Table 1:** Endoscope comparisons in older interventions WLE: White-light endoscope

Endoscope systems	WLE	Ultra-thin endoscope	Capsule endoscope
Endoscope diameter approx.	9-13 mm	5-6 mm	11 mm
Field of view	140-170 degrees	120-140 degrees	145-170 degrees
Camera resolution	High definition	Standard definition	256x256-512x512 pixels
Scope guidance	Four-way angulation	two- and four-way angulation	Passive or external magnetic steering
Advanced imaging	Yes	Yes	No
Sedation requirement	Yes	No	No
Biopsy capability	Yes	Supported in some	No

Newer interventions

*Light-Emitting Diode (LED)*

The newest technologies are LED optics or laser-based emission systems. They intensify the mucosal surface and vascular pattern of the morphology. A recently published manuscript evaluates linked color imaging (LCI) technology, which uses LED optics to increase the efficacy of mucosal inflammation diagnosis in patients with IBD [39]. LCI technology provides a newer approach for assessing mucosal surface inflammation of the colon and better clinical outcomes in IBD patients.

*Virtual Chromoendoscopy*

This imaging uses a filter instead of dyes to contrast the mucosa and the superficial vasculature for the mucosa. It involves Olympus (narrow-band imaging (NBI)), i-scan (Pentax), and Fuji and non-Fuji intelligent chromoendoscopy. The most common modality of virtual chromoendoscopy is NBI due to its easy availability, interpretation, and inter-observer agreement [40]. The neoplastic lesions of colonoscopy are 0.47 for chromoendoscopy, and 0.32 for NBI, which found a significant difference in neoplastic detection between NBI and chromoendoscopy. The use of NBI is recommended by the ACG guidelines for the surveillance of dysplasia [41]. The colonoscopy malignant lesions are 0.47 for chromoendoscopy and 0.32 for NBI, indicating a considerable difference in neoplastic identification between the two methods. The use of NBI is recommended by the ACG guidelines for the surveillance of dysplasia [41].

*Endopathology*

Confocal laser endomicroscopy (CLE) and endocytoscopy are two examples of endopathology. The mucosa is greatly magnified by these procedures. They are more used for the characterization of lesions. These techniques alter the tissue analysis's vascular pattern and cellular structure.

1. CLE: The histological alterations linked to the disease activity can be predicted by imaging. The assessment of the crypts and the fluorescein leakage shows a relation to the histological results [42]. There are different kinds of fluorescein, which are used as contrast agents. Fluorescein is administered intravenously, and vital dyes such as a rifle vine, tetracycline or crestless violet are applied topically. The uptake of fluorescein increases the contrast in the mucosal crypts, villi, and vascular structures. When this method was compared to WLE, some patients with normal mucosa had acute inflammation on histology; when this method was compared to patients without normal mucosa or with chronic inflammation, it revealed acute inflammation on histology. Patients with IBD who had a connection between cell shedding and local barrier failure underwent this diagnostic procedure [43]. It shows remission and relapse for the diagnosis of IBD. The disadvantage of this procedure is that it is not cost-effective, and a learning curve is required for the practical use of the community.

2. Endocytoscopy : Endocytoscopy is present in detecting and distinguishing the mucosal inflammatory cells and inflammatory disease activity [44]. It is used to assess patients' histological improvement without needing biopsy specimens [45]. The imaging is up to 150-fold optical magnification at the cellular level. Methylene blue, combined with crystal violet, visualizes nuclear and glandular patterns to increase the mucosal surface contrast [25]. It has recently been developed in conjunction with computed-assisted diagnosis (CAD), but it is seen that it can be used for invasive colorectal cancer and polyps. The classification of surface and vascular patterns in IBD patients will be represented by the CAD. The GIT lesions can also be seen at a cellular level for commercial endoscytoscopy [46-48]. This procedure differs from CLE as it provides ultra-high resolution.

*Molecular Imaging*

Molecular imaging applies genetic and molecular profiling of structures, antibodies, peptides, lectins, and nanoparticles [49]. The detection of early lesions is based on macroscopic and microscopic morphological changes with higher specificity. Molecular imaging has been widely used in conjunction with fluorescence imaging modalities and infrared imaging to suppress confounding tissue. Endoscopic imaging provides a wide field imaging limitation when used with fluorescence and gives a high specificity. The advantage of the approach is that the longitudinal study with no tissue removal is a safe approach [50].

Current application of AI in endoscopy

AI helps endoscopy depend on personal computer calculations that proceed as human minds do [51]. They respond to what they get as data and what they have realized when fabricated. The major guideline of this innovation is "machine learning" (ML) [52].

**Figure 1:**
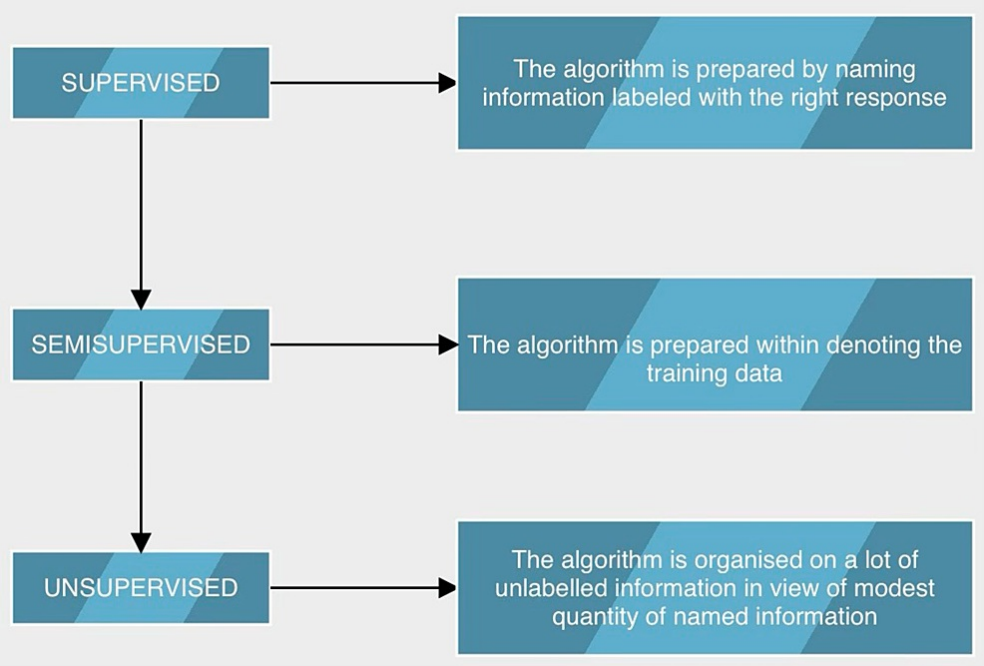
Algorithms involved in MLprocess. ML: Machine learning

There are various ML strategies (Figure 1) and one of the most well known is the utilization of artificial neural networks (ANN) [53]. ANN depends on various interconnected layers of calculations, which process information in a particular example and feed information so the framework can be prepared to do a particular undertaking [54]. Another diffuse ML strategy is the support vector machine (SVM), which is utilized for characterizing informational collections by making a line or plane to separate information into particular classes [55]. A development of ML is deep learning (DL): a complex, multilayer brain network design learns portrayals of information consequently by transforming the information data into various degrees of reflections [56,57]. A development of the less difficult ANN is the convolution neural network (CNN), propelled by the reaction of human visual cortex neurons to a particular upgrade and having the option to convolve the info and pass its outcome to the following layer [51].

AI in the detection of IBD

One of the primary uses of computer-based intelligence has been the endeavor to work with the determination of IBD along with the different analysis among CD and UC. In the model of Mossotto, three administered ML models were created using endoscopic information just, histological just, and consolidated endoscopic/histological findings [58]. Quénéhervé and partners attempted to plan a model to analyze IBD and lay out different conclusions between CD versus UC [59]. They put together their review with respect to CLE, which is a variation of light microscopy by which central laser brightening is joined with pinhole restricted discovery to mathematically dismiss out-of-center light [60].

AI for cancer in chronic IBD

Considering the expanded risk of creating colorectal cancer, reconnaissance colonoscopy assumes a significant part in the administration of UC [4]. Chromoendoscopy, which employs indigo carmine or methylene to more accurately define the superficial GI mucosa, is the highest quality level method for observing dysplasia [61]. The main experience is a case report of Maeda and partners where the Endo-BRAIN eye framework was tried for distinguishing dysplasia in a patient with well established UC [62]. This framework can distinguish colorectal sores with high precision in the overall public, yet for this situation it is demonstrated to help endoscopists in the recognizable proof of UC-related dysplasia, which is generally difficult to identify because of its level appearance and hazy limits [62].

## Conclusions

The advanced endoscopic imaging techniques have increased the recommendation of newer techniques for defined imaging. High-definition endoscopes should be used with traditional dye spraying methods to enhance the endoscopy system. The principle lies in visualizing the mucosal vascular pattern of the morphology. GIT endoscopy can be categorized accordingly as diagnostic and therapeutic. The newer molecular imaging and endoscopic interventions are under clinical evaluation, which can lead to detection and improve accuracy for the GIT lesions. It can also improve the healthcare communities with newer opportunities. Newer interventions are better than old ones due to cost effectiveness, improved diagnostic criteria, and better visualization techniques. A learning curve and the need for training and experience to evaluate clinical data utilizing optical imaging methods are two drawbacks of more recent therapies. While adaption of newer technologies treating and assessing diseases would be comparatively decreased, complications would be present. AI is a foundation upheaval in endoscopy. In the field of IBD, its essential applications are giving extraordinary outcomes in the conclusion and organizing of the illness. In this field of medication, where the ongoing technique is the treat-to-target technique and where treatment headings are directed by endoscopic reduction, a reasonable and explicit device capable of conquering human limits could address an extraordinary partner. High-performing analytic guides with low changeability are valuable in the location and normalization of results and in the objectives' evaluation. Also, in the event that mucosal recuperation could be seen as a practical objective, an idea that pushes ahead and takes to the super the past thought is sickness freedom. With regards to AI and large information, a future viewpoint is the production of calculations for finding and checking of IBD put together with respect to endoscopic, yet in addition to clinical and histological information to have a total outline of all illness highlights.
